# The impact of protected area governance and management capacity on ecosystem function in Central America

**DOI:** 10.1371/journal.pone.0205964

**Published:** 2018-10-18

**Authors:** Carlos L. Muñoz Brenes, Kelly W. Jones, Peter Schlesinger, Juan Robalino, Lee Vierling

**Affiliations:** 1 Department of Natural Resources and Society, University of Idaho, Moscow, Idaho, United States of America; 2 Economics and Environment for Development Research Program–EfD Central America, Tropical Agricultural Research and Higher Education Center (CATIE), Turrialba, Costa Rica; 3 Human Dimensions of Natural Resources, Colorado State University, Fort Collins, Colorado, United States of America; 4 Postgraduate School, Tropical Agricultural Research and Higher Education Center (CATIE), Turrialba, Costa Rica; 5 School of Economics, Universidad de Costa Rica, San Pedro, San José, Costa Rica; Universidade de Brasilia, BRAZIL

## Abstract

Protected areas (PAs) are a prominent approach to maintaining and enhancing biodiversity and ecosystem services. A critical question for safeguarding these resources is how PA governance processes and management structures influence their effectiveness. We conduct an impact evaluation of 12 PAs in three Central American countries to assess how processes in management restrictions, management capacity, and decentralization affect the annual change in the satellite-derived Normalized Difference Vegetation Index (NDVI). NDVI varies with greenness that relates to plant production, biomass, and important ecosystem functions related to biodiversity and ecosystem services such as water quality and carbon storage. Any loss of vegetation cover in the form of deforestation or degradation would show up as a decrease in NDVI values over time and gains in vegetation cover and regeneration as an increase in NDVI values. Management restriction categories are based on international classifications of strict versus multiple-use PAs, and capacity and decentralization categories are based on key informant interviews of PA managers. We use matching to create a counterfactual of non-protected observations and a matching estimator and regression to estimate treatment effects of each sub-sample. On average, strict and multiple-use PAs have a significant and positive effect on NDVI compared to non-protected land uses. Both high and low decentralized PAs also positively affect NDVI. High capacity PAs have a positive and significant effect on NDVI, while low capacity PAs have a negative effect on NDVI. Our findings advance knowledge on how governance and management influence PA effectiveness and suggest that capacity may be more important than governance type or management restrictions in maintaining and enhancing NDVI. This paper also provides a guide for future studies to incorporate measures of PA governance and management into impact evaluations.

## Introduction

Protected areas (PAs) are critical to global conservation goals. They are created to protect and enhance biodiversity and ecosystem services. Many PAs also contain important features of geological and ecological processes, as well as cultural values [1]. About 15.5% of the earth’s terrestrial surface and 3.4% of the global ocean area have been set aside and designated as PA under some type of management category—and there are international commitments to expand this area to 17% of terrestrial and 10% of marine areas under Aichi Biodiversity Target 11 [2]. Despite global commitments to establish more PAs, poor management, lack of funding, unenforced legislation, and outside threats, hinder their effectiveness [3, 4]. As a result, deforestation and biodiversity loss continue at disturbingly high rates within many PAs [5, 6].

An increasing number of counterfactual evaluations shed light on PA effectiveness and the factors associated with impact [6]. A counterfactual is what the outcome would have been like in the absence of the intervention [7]. A global impact evaluation of forested PAs found that 75% of the sampled countries experienced reduced deforestation due to PAs, when they were compared to similar areas without protection [8]. National and regional impact evaluations have also found positive impacts of PAs on avoiding deforestation, ranging from 1–2%-point reductions in deforestation to as high as 25%-point reductions [6, 9]. Shafer [10] suggests that allowed land uses in the PA buffer zone and monitoring of compliance affects the success of protection in PA core sites. A general conclusion from these studies is that the amount and types of threats a PA faces affect the outcome from protection, with PAs located in areas with higher prevalence of threats having to avoid more deforestation, and thus showing a larger counterfactual impact on reducing deforestation.

There is often a correlation between *de jure* PA management restrictions and PA threats based on the location and management objectives of PAs [11, 12]. The International Union for Conservation of Nature (IUCN) groups PAs in six management restriction categories, ranging from strictly protected sites with highly restricted access to people, to multiple-use areas that have more open access and have fewer restrictions and integrate sustainable resource use with conservation [13]. A common way to test PA impacts is to consider whether the PA is designated for strict protection, or if sustainable uses are allowed [11, 12, 14–18]. Findings are mixed about the influence of these restrictions, with several studies finding higher positive impacts associated with strict PAs [15, 19] and others finding that multiple-use areas have higher positive impact [16, 17]. Using *de jure* PA management restrictions does not control for *de facto* enforcement or the management capacity of a particular PA [12].

There is increasing attention to understanding how a governance regime affects PA impacts [11, 20, 21]. Macura and Secco [22] define governance of PAs based on the type of actors involved, their responsibility, accountability, level of power sharing and type of knowledge, and several PA impact evaluations classify PA governance regime as either state, community, private, or co-governed. Some 5.4 billion ha (86%) of global forest is state controlled and regulated by public governance structures, about 10% is under private ownership, and 4% in other forms of management such as communal lands [23]. Reflecting this, most PA impact evaluations have focused on understanding state or community governance effectiveness, with fewer studies including multiple governance types in the same study, or examining private or co-governed PAs [6, 9, 22]. Common PA governance effectiveness measurements we found in the literature relate to forest or land cover change, deforestation, environmental integrity, biodiversity status, or regional development as conservation outcomes [24, 25]. The results of these studies indicate that both state and community-managed PAs can successfully avoid deforestation and conserve forest habitat, with PA effectiveness varying by PA location (e.g., far from roads) and country. While there can be correlation between governance regime and management restriction categories—for example, community PAs may be more likely to be designated multiple-use areas—the relationship is not one-to-one [22]. However, the complexity of PA governance cannot be analyzed by looking at a single characteristic or indicator [26].

Decentralization is a component of governance regimes [22]. Decentralization occurs when a centralized governance regime, with management and decision making of a PA by the central state, is changed so that local state actors or non-state actors, such as indigenous communities, are the managers and decision makers; the latter case is also classified as communal governance regime [27, 28]. There has been an increase at the global scale in decentralized and co-managed PAs over the past few decades, mainly driven by new legislation and policy and the influence of global forces [29]. The argument for decentralization is that greater local engagement leads to better outcomes because there is more acceptance of decisions and they are informed by local knowledge [30]. Proponents of decentralization see it as an opportunity for greater local participation and control in the governance process [31, 32]. Others, however, have pointed to potential drawbacks, including cases where decentralization efforts do not increase the powers of local authorities or peoples [33], or where greater participation does not necessarily lead to better conservation outcomes; for example, locals devise rules that allow forest conversion or greater access to resources and benefits detrimental to longer term resource sustainability [31, 34–37]. Few PA evaluations have tested whether the level of decentralization of a PA influences conservation outcomes [38].

The variation in PA impacts across management restrictions and governance regime suggests that there are important contextual, or moderating factors affecting how these designations influence conservation outcomes. One likely moderating influence is the management capacity of a PA. Governance is not management—governance is the set of processes and institutions that help define management goals. Management is about implementing the practical measures to achieve those goals, its aim is to improve outcomes directly while governance seeks to define what good outcomes are and sets the decision-making process of management activities to achieve those goals [25, 39]. Management capacity is about having the means to accomplish management objectives as well as the ability to make effective decisions [32]. One study using self-reported data from multiple PAs suggests that management capacity—for enforcement, boundary demarcation, and direct compensation to local communities—and conservation goals are correlated [40]. The most comprehensive assessment of PA management capacity is the Global Database for Protected Area Management Effectiveness (GD-PAME), which tracks indicators such as existence of a management plan, law enforcement activities, PA budget and staff, and threats [41]. A handful of studies have assessed whether a correlation exists between GD-PAME indicators and changes in forest cover or fires in Brazil and the Amazon Basin; however, no such correlation has been found [18, 19, 42]. A study from Mexico does find that well-funded PAs have larger impacts on conservation outcomes than poorly-funded PAs [16].

Our study adds to the small but growing set of evaluations rigorously assessing the relationship between PA governance, management, and conservation impacts. The goal of this study is to test how governance and management affect the ability of 12 PAs in the Trifinio Region of Central America to enhance and maintain vegetation cover estimated by the Normalized Difference Vegetation Index (NDVI) derived from satellite imagery. NDVI varies with greenness that relates to plant production, biomass, and important ecosystem functions necessary for biodiversity and ecosystem services such as water quality and carbon storage. Due to the fact that NDVI is calculated using common spectral bands in red and near infrared wavelengths, researchers worldwide use NDVI change as an indicator of changes in vegetation density and cover, and as a proxy for deforestation [43, 44]. Thus, remotely sensed NDVI measures combined with qualitative data on governance and management capacity is a practical way to differentiate between natural variation in ecosystem function and human induced variation due to PA creation [45]. First, we examine how management restrictions, standardized according to the IUCN classification, correlate with NDVI change. Second, based on key informant interviews we construct measures of (1) level of decentralization of state PAs in decision-making and co-management and (2) management capacity, and test the impact of these classifications on NDVI variation. We use matching to pre-process our data and a matching estimator and regression to measure the impact of PAs on NDVI change over a 30-year period. While the first objective is commonly performed in impact evaluations of PAs, the second objective provides measures of PA governance and management capacity, constructed from primary data that few studies have linked to PA outcomes [22, 38]. Our findings shed light on the factors that influence PA effectiveness and our combination of qualitative data with impact evaluation methodology provides a template that can help advance causal theories of change for how PA governance and management influence conservation outcomes.

## Methods

### Study area

Our study takes place in the Trifinio Region of Central America. This tri-national region has an area of 7541 km^2^ in southern Guatemala (45%), western Honduras (40%), and northern El Salvador (15%). Trifinio became a political administrative unit by international agreement after the 1987 Peace Accords, which followed more than 40 years of civil wars. Population has grown considerably since 1987, with estimates showing that the region has almost doubled from 572,000 to nearly 900,000 people between 1987–2011 [46]. High rates of poverty exist in the region, with 87% of people reported as poor and 53% as extremely poor [47].

The Trifinio Region was specifically created to promote the conservation and sustainable use of natural resources as well as the protection of cultural heritage. Sixteen PAs have been created in Trifinio, including a transboundary PA aimed at safeguarding water resources for nearly three million people living downstream [48, 49]. Our study focuses on 12 state-managed forest PAs that were established as of 1987—five PAs are in Guatemala, six in Honduras and one in El Salvador.

A recent land cover assessment of the region found a 1.5% annual forest conversion rate between 1986 and 2010 [50]. The rate of conversion was higher in 2001–2010, at 2.6% per year, compared to <1% per year in 1986–2001, indicating increasing pressures on forests, and presumably, PAs. Similar to other tropical regions, this forest loss is associated with agriculture and pasture expansion [50].

### Protected area governance and management classifications

The 12 forest PAs in Trifinio are classified into two management restriction levels following the IUCN classification system—strict (Categories I-II) and multiple-use (Categories III-IV). PAs in IUCN categories I and II are mainly created for science, wilderness protection, ecosystem protection, and recreation, and PAs in IUCN categories III-IV are established largely for conservation of specific natural features or sustainable use of natural resources. We used the reported IUCN category from the World Database on Protected Areas, and for unreported PAs, we identify an equivalent IUCN classification based on stated management objectives [51].

**Fig 1:**
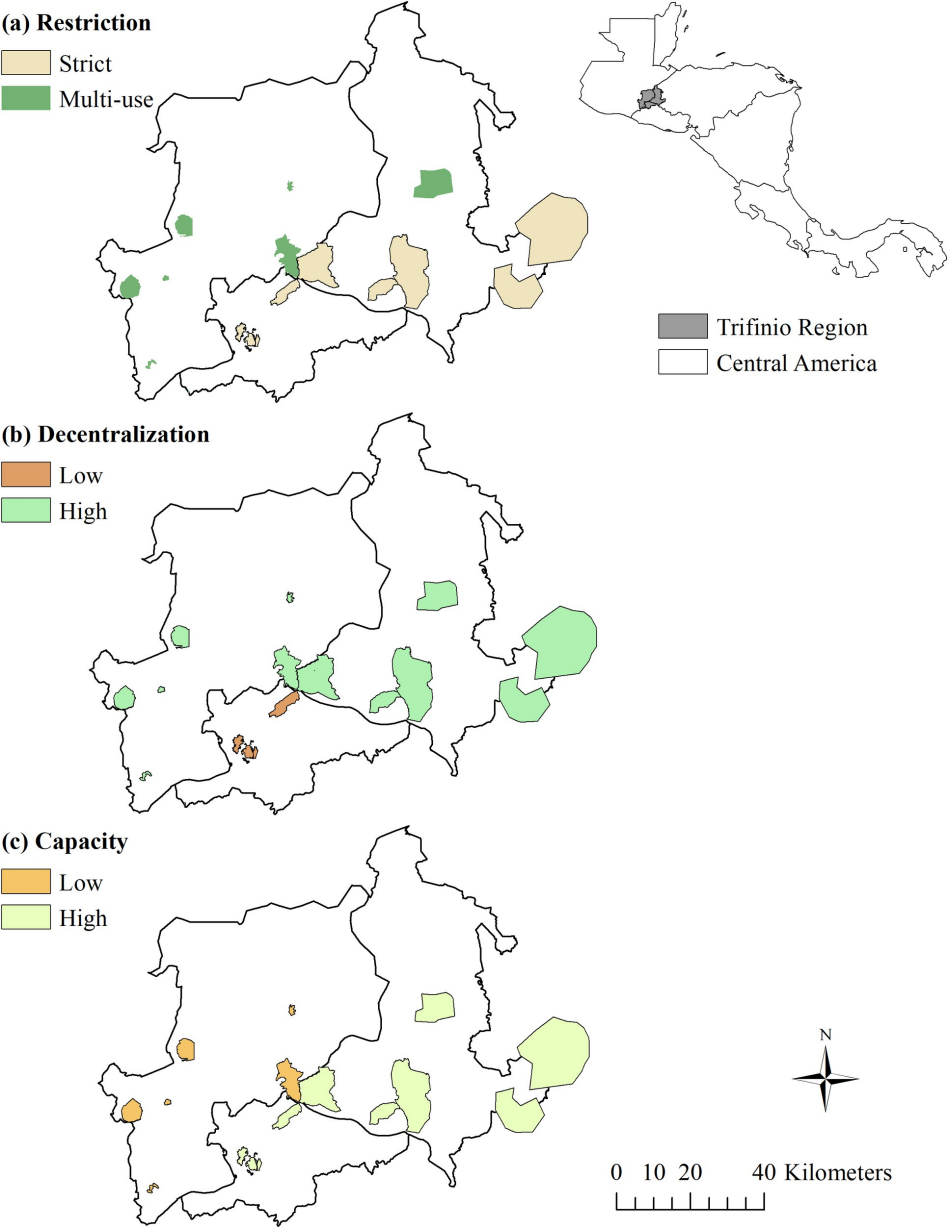
Protected areas in Trifinio Central America. Classification of PAs by levels of (a) restriction, (b) decentralization, and (c) management capacity. The Central America map shows the location of the study area in gray.

We use primary data to construct PA classifications of level of decentralization and management capacity (Fig 1). We conducted nine key informant interviews with PA staff and five interviews with professionals working on PAs in the Trifinio Region. A semi-structured questionnaire was used to collect information on governance structures, decision-making processes, management capacity, and conservation outcomes for each of the 12 PAs; key elements we used to assess decentralization and management capacity are summarized in Table 1. The research project (project 15–918) was approved by the Institutional Review Board at the University of Idaho, certified as exempt under category 2 at 45 CFR 46.101(b)(2). Oral informed consent was obtained from all interviewed participants whom were informed that participation in the interview was confidential and voluntary, had the right to stop at any time, did not have to answer a question should they wish not to do so, and that all information collected will be treated confidentially and only for research purposes. To the best of our knowledge we complied with relevant national regulations and laws applying to foreign researchers in Guatemala, Honduras, and El Salvador and specific permits or approvals were not required to conduct our research. All meetings took place in the Trifinio countries between September and October 2015 and were voice recorded and transcribed. Interview time was between one and two hours.

**Table 1:** Factors used to classify PAs by decentralization and management capacity.

Factors related to Decentralization	Factors related to Management Capacity
Entity that holds responsibility for the PA (e.g., Secretary or Ministry, local organization as co-manager)	Existence of written management plan or annual operations plan
Authority responsible for appointing the director or person responsible for the PA (e.g., central government, local authority)	Main sources of funding for PA
Ways this person makes management decisions for PA (e.g., in consultation, dependency on centralized authority)	PA budget fluctuations
Political interference and transparency in decision making	Number of staff working for the PA
The number of stakeholders participating or involved in decision-making and management activities and coordination	Relevant data about PA is generated and available (e.g., biodiversity inventories, visitation, status of infrastructure, boundaries)
Frequency of interaction or meetings with external relevant actors, communication, and opportunity for feedback	Priority distribution of PA budget (e.g., staff, equipment and infrastructure, research)
Existence of co-management agreements	Allege illegal activities in PA lead to sanctions

We use cluster analysis combined with expert opinion from our interviews as a systematic way to generate the groups of PAs into the different categories in the decentralization levels and the capacity levels [52, 53]. Cluster analysis identifies patterns, or clusters, in data, based on similarities (additional details on cluster method and procedures used are found in S1 Appendix). We first use a subset of interview questions and a series of Likert-scale questions about decentralization and management capacity for clustering (some of the governance elements related to decentralization and capacity used to classify PAs are in Table 1 and interview question themes are reported in S1 Table). The results of the cluster analysis were then reviewed by members of our study team with expert knowledge on the region, who determined that the grouping of PAs into sub-categories fit their knowledge of the PAs. The total number of pixels and PAs in our study that fall within each sub-group classification by decentralization, management restriction, and management capacity are in Table 2. We treat the three classifications in Table 2 as independent of one another in this study, with the primary goal of testing the relationship between each classification and NDVI change outcomes.

**Table 2:** Number of PAs and sampled pixels by governance and management classification.

	Management restriction		Management capacity		Decentralization	
	Strict	Multiple-use	High	Low	High	Low
PAs	6	6	7	5	4	8
NDVI pixels (30 m by 30 m)	11,612	10,288	17,527	4,373	8,835	13,065

### Dependent and independent variables

NDVI can be used to derive gross primary plant production, biomass, seasonality in productivity, and phenological activity [45, 54, 55]. Most impact evaluations of PAs have used avoided deforestation as their outcome, which requires land cover classification. While we chose to use NDVI, or greenness-based land cover, the spectral similarity from satellite mapping between coffee-based agroforestry systems, forests, and other cover types in our study area may lead to over-classification of forests [56, 57]. Despite this limitation NDVI is an important ecological measure of PA effectiveness, with higher NDVI values generally associated with greater leaf area and more ecosystem function, which often indicates higher levels of biodiversity and provision of ecosystem services such as water quality and carbon storage. NDVI change is used worldwide as a practical way to measure human induced variation in land covert (e.g., deforestation or increases in forest covert by conversation interventions) [43–45]. The use of NDVI is also an efficient way to estimate increase in disturbances from decline in ecosystem function and productivity or land degradation [58]. A recent assessment of global PAs found strong correlations between PAs and maintenance of NDVI [59].

**Fig 2:**
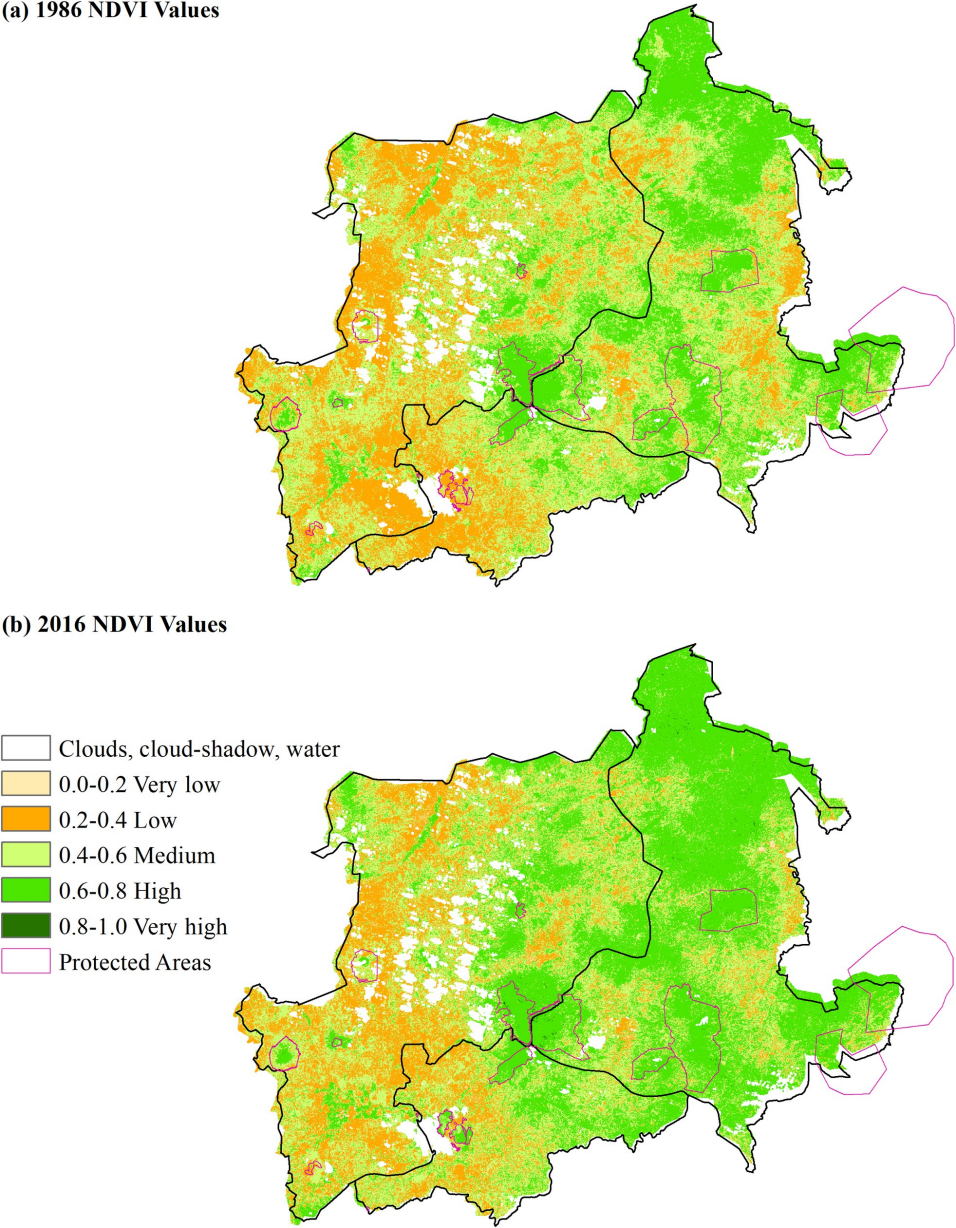
Variation in NDVI measures in Trifinio between 1986 and 2016. NDVI variation inside and outside PAs in Trifinio between 1986 (a) and 2016 (b). Pixels within PA polygons of selected forest PAs inside the Trifinio region were sampled as treatment units all control pixels are outside PAs. Clouds, cloud-shadow, and water pixels (Class 0) were masked out prior to cutting the rasters. The raster maps were reclassed to show variation in NDVI values as follows: Class 1 is >0 and <0.2, Class 2 is > 0.2 and < 0.4, Class 3 is >0.4 and <0.6, Class 4 is >0.6 and < 0.8, and Class 5 is >0.8 and < 1.0. There are no 1.0s in the raster database. A 3x3 mode filter is used on the reclassed pixels to reduce single vegetated polygons to approximately a hectare minimum. The map projection is UTM16N.

Medium resolution remotely sensed Landsat images (30 m pixels) were acquired from the USGS Earth Resources and Observation Science Center (USGS/EROS). The area of Trifinio comprises a single Landsat scene (World Reference System (WRS) Path/Row 19/50). We developed least-cloud, NDVI values over 30 years in seven epochs, spaced at approximately five-year intervals, starting in 1986 and ending in 2016 (more details on remote sensing analysis are in S2 Appendix). Cloud cover is high in the region, and least cloud data are only accessible during the dry season (between January and March), which can affect NDVI values due to low rainfall and crop irrigation. While this can lead to measurement error, this should not be correlated with the placement of PAs, and therefore should not bias our estimates of PA effectiveness. We sampled pixels that were free of clouds, shadows, and water bodies, both inside and outside of PAs in Trifinio (Fig 2).

NDVI is a continuous measure and we scale it between 0 (no vegetation) and 1 (maximum/saturating levels of greenness). NDVI values in the range of 0.2 to 0.5 generally correspond to sparser vegetation row crops, shrubs and grasslands, whereas dense vegetation such as temperate and tropical forests show higher values between 0.6 to 0.9; lesser NDVI values correspond to dry vegetation, barren rock, sand, and built-up areas [60]. Higher NDVI values are considered better outcomes for PAs in terms of ecosystem functioning and biodiversity. Any loss of vegetation cover or degradation would show up as a decrease in NDVI change over time and gains in vegetation cover and regeneration as an increase in NDVI change. While saturation in NDVI values (i.e. reduced change in NDVI with increased greenness) can occur at high levels of greenness, visual analyses of higher resolution images revealed that differences between forest/coffee and other land use types are large enough during the dry season so as to minimize the impact of saturation effects on the analysis [57, 61].

We develop two dependent variables with these data: (1) mean NDVI value for 1986–2016, averaging NDVI values across all seven epochs, and (2) annual change of NDVI values between 1986–2016. With the first measure (mean NDVI), we test whether PAs have maintained higher greenness levels, and thus ecosystem functioning, compared to areas outside protection (i.e., “maintenance” of NDVI). With the second measure, we test the annual change at which PAs gain or lose vegetation greenness since designation, and thus whether we can attribute increases (or decreases) in ecosystem functioning to PAs (i.e., “enhancement”, or deforestation and “degradation”, accordingly). Aside from natural phenology changes, vegetation clearing or degradation within a pixel would decrease NDVI values [44].

For each pixel in our data set we sample several spatial variables expected to be correlated with designation as a PA and greenness values, to control for the non-random location of where PAs are placed on the landscape [62–64]. These include baseline NDVI, elevation, slope, and distance to roads, municipality capitals and country capitals. Several studies have demonstrated a strong effect of these covariates on assignment to protection status and deforestation rates in PA evaluations [15, 62, 65, 66]. One study reports more forest loss in remote areas of Trifinio and at lower elevations [50].

### Analysis

A counterfactual evaluation estimates the mean difference between the outcome with protection and the outcome without protection [7]. Since this counterfactual is never actually observed, the researcher must create one. We use the quasi-experimental evaluation method of matching to control for the non-random allocation of protection and create a control group that is as similar as possible to PA observations [67]. Matching involves pairing treatment and comparison units that are similar in terms of their observable characteristics [68]. After units in the comparison groups are matched to a treatment unit, the unmatched comparison units are discarded and are not directly used in estimating the treatment effect [69].

We use propensity score matching (PSM) to select the best set of control observations [70]. A propensity score (PS) is estimated for all protected and non-protected pixels; the PS is the probability of exposure to the treatment status conditional on observed characteristics. Treated and untreated observations with similar PS values are matched to create the new, trimmed dataset. We select only one match (the nearest neighbor) for each PA pixel and match without replacement. We limit the distance between PS values using a caliper and the common support function, which is recommended to improve the quality of the matches [71–74]. PSM is done for each sub-group of PAs and the full set of unprotected observations (Table 2); thus, the best set of control observations is match to each sub-group of PAs. We check covariate balance after matching using quantile-quantile plots and normalized differences in mean values [67]. The balance improves significantly in all sub-groups after matching (S3 Table) and all covariates pass the rule of thumb of normalized differences in means less than 0.25 [73, 75–77].

After trimming the sample to the best control group, the treatment effect can be estimated by the differences in means across the matched treatment and control groups. However, because matching in finite samples is never exact, an adjustment procedure can be used to correct for remaining bias [78, 79] or post-matching regression can be used [80]. We implemented both methods with the trimmed sample of pixels, controlling for the set of covariates listed above and estimating robust standard errors. We implement the bias-adjustment using the nearest neighbor matching estimator using one and five nearest neighbors and two different matching metrics, Mahalanobis distance metric and the inverse diagonal sample covariate metric, to check for consistency in estimates. We implement post-matching regression on the trimmed sample of pixels using linear ordinary least squares (OLS) regression. We report the average treatment effect on the treated (ATT); the ATT is conditional to only those units receiving the treatment [67, 81]. We checked the sensitivity of our treatment effect estimates using Rosenbaum bounds, which determines how strongly hidden bias from an unmeasured variable must influence the selection process to undermine the implications of the matching analysis [82].

## Results

We randomly sampled close to 130,000 pixels from outside of PAs, for a total dataset of over 150,000 pixels when combined with the sampled pixels in each PA sub-group (Table 2). Average NDVI values are higher for all sub-groups of PAs compared to the non-protected average NDVI value of 0.56 (Table 3 and S1 Fig). A graph of NDVI over the seven epochs shows increasing greenness inside and outside of PAs in the Trifinio Region since 1986 (S1 Fig). This is reflected in the positive annual changes in NDVI ranging between 0.2–0.4 in all sub-groups of the data (Table 3). Mean NDVI is most similar for non-protected pixels and low capacity PAs but across all sub-groups of PAs greenness increased over time (Table 3 and S1 Fig).

**Table 3:** Summary statistics.

Variable	Not PA	Strict PA	Multiple-use PA	High Capacity	Low Capacity	High Decentralization	Low Decentralization
Annual change in NDVI 1986–2016 (%)	0.27	0.31	0.34	0.36	0.18	0.32	0.33
	(0.39)	(0.34)	(0.42)	(0.36)	(0.40)	(0.31)	(0.42)
Mean NDVI 1986–2016	0.56	0.72	0.66	0.72	0.59	0.75	0.66
	(0.14)	(0.10)	(0.14)	(0.10)	(0.17)	(0.09)	(0.13)
Elevation (masl)	1036.95	1914.41	1629.12	1846.20	1513.81	2022.62	1616.71
	(359.40)	(303.57)	(295.24)	(297.39)	(327.40)	(134.48)	(327.58)
Slope (%)	29.65	39.94	42.40	40.43	44.81	41.46	41.13
	(18.79)	(20.61)	(19.36)	(19.82)	(20.91)	(21.62)	(18.90)
Distance to road (km)	1.87	3.14	1.86	3.08	1.92	4.12	1.93
	(1.92)	(2.60)	(1.06)	(2.76)	(0.95)	(3.23)	(1.26)
Distance to municipal capital (km)	8.69	9.52	8.41	8.91	7.23	8.51	8.65
	(8.18)	(3.95)	(2.61)	(4.06)	(2.25)	(4.92)	(2.78)
Distance to country capital (km)	166.22	175.50	159.46	185.25	96.55	186.57	154.69
	(58.59)	(42.70)	(55.42)	(38.05)	(17.65)	(27.72)	(57.12)
Observations	128,355	11,612	10,288	17,527	4,373	8,835	13,065

Note: Mean values with standard errors in parentheses.

PA characteristics vary across each sub-group (Table 3). Strict PAs have higher average NDVI values than multiple-use PAs, are at higher elevations, and are much more remote. These attributes suggest lower threats to ecosystem change, deforestation, and degradation for strict PAs than multiple-use PAs. High capacity PAs have higher average NDVI values than low capacity PAs, are at higher elevations and are farther from roads. Average NDVI is higher for PAs classified as high versus low decentralization, and PAs ranked as high decentralization have higher elevations and are farther from roads.

**Fig 3:**
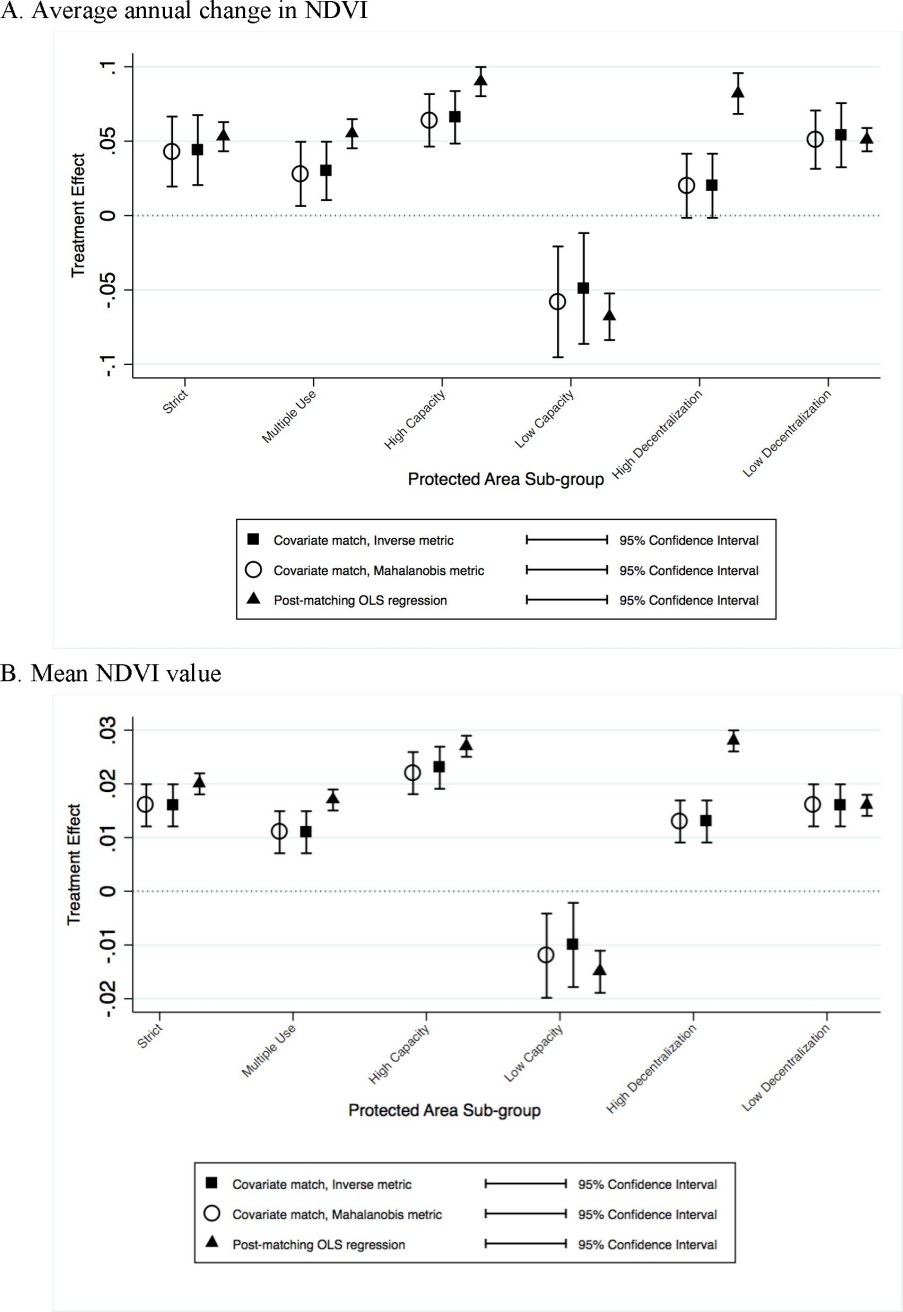
Estimated impacts of PAs on NDVI outcomes showing mean value and 95% confidence intervals.

Post-matching regression and nearest neighbor matching estimators suggest that strict PAs have a positive and statistically significant impact on annual change in NDVI and mean NDVI for the 30-year period (Table 4). Strict PAs have an average annual change of 4–5%-points higher, and a mean NDVI value of 2%-points higher than similar non-protected pixels. The average treatment effect across estimators are statistically similar (Fig 3). Multiple-use PAs, which are located in areas with higher prevalence of threats, have a statistically significant change in NDVI between 3–6% -points higher than non-protected pixels. They also have higher mean NDVI values, about 1–2%-points, than similar non-protected areas. The different estimators result in statistically similar average treatment effects (Fig 3). Thus, both strict and multiple-use management restrictions in the Trifinio Region maintain and enhance greenness relative to similar areas outside of protection. The full post-matching regression output can be found in (S2 Table).

**Table 4:** Impact of PAs on NDVI outcomes by governance and management sub-groups.

Sub-group of PAs	Average annual change in NDVI 1986–2016 (%)			Mean NDVI value 1986–2016		
	“Enhance NDVI”			“Maintain NDVI”		
	Matching estimator		Post matching	Matching estimator		Post matching
	Mahalanobis metric	Inverse metric	OLS regression	Mahalanobis metric	Inverse metric	OLS regression
Strict	0.043***	0.044***	0.053***	0.016***	0.016***	0.020***
	(0.012)	(0.012)	(0.005)	(0.002)	(0.002)	(0.001)
Observations	14,500	14,500	14,500	14,500	14,500	14,500
Multiple-use	0.028***	0.030***	0.055***	0.011***	0.011***	0.017***
	(0.011)	(0.010)	(0.005)	(0.002)	(0.002)	(0.001)
Observations	20,292	20,292	20,292	20,292	20,292	20,292
High Capacity	0.064***	0.066***	0.090***	0.022***	0.023***	0.027***
	(0.009)	(0.009)	(0.005)	(0.002)	(0.002)	(0.001)
Observations	22,714	22,714	22,714	22,714	22,714	22,714
Low Capacity	-0.058***	-0.049***	-0.068***	-0.012***	-0.010***	-0.015***
	(0.019)	(0.019)	(0.008)	(0.004)	(0.004)	(0.002)
Observations	6,902	6,902	6,902	6,902	6,902	6,902
High Decentralization	0.020*	0.020*	0.082***	0.013***	0.013***	0.028***
	(0.011)	(0.011)	(0.007)	(0.002)	(0.002)	(0.001)
Observations	7,784	7,784	7,784	7,784	7,784	7,784
Low Decentralization	0.051***	0.054***	0.051***	0.016***	0.016***	0.016***
	(0.010)	(0.011)	(0.004)	(0.002)	(0.002)	(0.001)
Observations	25,384	25,384	25,384	25,384	25,384	25,384

Note: Robust standard errors in parentheses. All estimators use a trimmed sample of PA and not PA observations based on propensity score matching (PSM). PSM is done for each sub-group of PAs to select a unique control group for that set of treatment observations. Following PSM, nearest neighbor matching and OLS regression is used to adjust for remaining differences in observables [83]. Matching includes the following covariates: baseline NDVI, elevation, slope, distance to roads, municipal capital and country capital. Matching results are reported for one nearest neighbor; matching on five nearest neighbors produced qualitatively similar results.

* p<0.10

** p<0.05

*** p<0.01

There is not a large difference in high versus low decentralization status PAs in terms of safeguarding biodiversity and ecosystem function; both types of PAs have positive and statistically significant effects on NDVI (Table 4). High decentralized PAs maintain greenness with mean NDVI values between 1–3%-points higher than outside PAs; their impact on enhancing NDVI ranges between 2–8%-points. These point estimates do vary across estimators, with significant differences between those using nearest neighbor matching and those using post-matching regression (Fig 3). More centralized PAs experience changes in greenness around 5%-points, and have mean NDVI values that are 2%-points higher than unprotected areas. These results are statistically similar across estimators (Fig 3). Therefore, having more centralized governance arrangements of the state-run forest PAs and having more decentralized participatory control both achieve conservation outcomes in the Trifinio Region.

High capacity PAs have a positive and statistically significant change in greenness of 6–9%-points, and a mean NDVI value of 2–3%-points higher, compared to similar control observations. These point estimates are similar across estimators (Fig 3). Low capacity PAs, however, experience a loss in greenness over the 30-year period compared to similar areas outside PAs, with a decrease of 5–7%-points. Low capacity PAs also do not maintain their greenness, with mean NDVI decreasing by 1–2%-points compared to areas outside of protection. The three estimators result in similar average treatment effects (Fig 3). Across both ecosystem function measures, higher capacity PAs result in better conservation outcomes than low capacity PAs.

Looking at all sub-groups of PAs we find that the average treatment effect sizes are statistically similar for strict, multiple-use, high decentralization, and low decentralization PAs (Fig 3). The treatment effect for high capacity PAs is slightly higher than other sub-groups, and low capacity PAs have a much lower treatment effect than any of the other sub-groups. These results should be interpreted carefully, however, given the small number of PAs within each sub-group. Additionally, the Rosenbaum bounds test for sensitivity to unobserved factors or hidden bias did suggest sensitivity to small changes in hidden bias. The latter test cannot state whether there is hidden bias affecting our estimates, but does suggest that the results in Table 3 are sensitive to deviations from the identifying assumption of matching of unconfoundedness [82].

## Discussion

Effectiveness in avoiding forest, biodiversity and habitat loss or degradation from protection is not guaranteed by any form of PA governance or management structure [20]. In this study, we examine how governance and management affect ecosystem function in the Trifinio Region of Central America with a broader goal of adding to the state of knowledge regarding PA effectiveness worldwide. In addition to providing impact estimates that can be compared to other studies that examine heterogeneity across management restrictions, this study utilizes primary data to construct measures of decentralization and management capacity and estimates how these characteristics influence conservation outcomes. While our findings should be interpreted as correlative, versus causal, given our sample size, they do shed light on the types of governance and management characteristics that future PA impact evaluations should strive to incorporate in their studies to advance knowledge on how PAs influence conservation outcomes, in addition to whether PAs have an impact.

We find that strict and multiple-use PAs are achieving conservation outcomes in the Trifinio Region. Studies in Brazil [20, 84], Bolivia, Indonesia, Thailand [12], and Russia [15] suggest that strict PAs are more effective than multiple-use areas, while results in Brazil [11], Guatemala [17] and Mexico [16] find that multiple-use PAs have larger conservation impacts than strict PAs. These studies attribute differences in effectiveness to location and enforcement of management restrictions, e.g., allowable sustainable uses within multiple-use areas. In the Trifinio Region, multiple-use PAs are located at lower elevations, closer to roads, and closer to markets, than strict PAs—this is similar to other areas, with multiple-use PAs more likely to be located in higher threat locations than strict PAs. Multiple-use PAs do appear to block the higher prevalence of threats they face in the Trifinio Region, but strict PAs, despite experiencing less pressure, have also maintained and enhanced NDVI by avoiding deforestation.

While other PA impact evaluations have compared governance regimes, few have compared whether decentralization within the same management type influences conservation outcomes. Our results suggest that decentralization *per se* is not strongly correlated with conservation effectiveness in the Trifinio Region. Advocates of decentralization suggest that more local involvement will lead to better conservation outcomes, but others have shown that local authorities can have incentives to manage natural resources unsustainably and may not have the resources to be successful at managing local resources. Both centralized and decentralized PAs are leading to positive conservation outcomes in Trifinio; our findings show decentralization does not lead to negative outcomes. This should be reassuring for proponents of decentralization, and suggests that which actors make the management decisions may not be as important as other factors, such as the decisions being made, or the management capacity to enforce and carry out those objectives.

In the Trifinio Region PAs with higher capacity appear more successful at achieving conservation outcomes than lower capacity PAs. This finding supports global investments in tracking and enhancing management capacity within PAs [40, 41], but is one of the few impact evaluations that have found a positive correlation between capacity and conservation outcomes. Previous studies have used PA budget to measure PA capacity [16], however, clustering on several variables that capture multiple dimensions of capacity, including use of management plans, boundary demarcation, enforcement, staff, budget, and equipment (Table 1 and S1 Table), may provide a more robust measure of PA capacity. While our study focuses specifically on measuring capacity within the PA, national-level institutions and enforcement processes outside the control of the PA will also influence conservation effectiveness.

Though our focus was on testing the influence of governance and management on conservation effectiveness, an interesting alternative would be to test how the combination of factors, e.g., strict PAs with high decentralization or high capacity, affect conservation outcomes. Our sample size is too small and lacks the variation needed to test across combinations of these factors. Better causal theories of change for PA governance and management are needed, and we highlight some plausible connections that future studies could explore. In our sample of PAs, we observe that management restrictions and capacity are positively correlated, with strict PAs more likely to have high capacity. Higher PA capacity within strict PAs could be caused by different factors. For example, governments may be more willing to provide resources to PAs where conservation is the primary objective, there may be more tourism visits to these PAs, which may or may not be in detriment of conservation goals [25], or international agencies may provide more funding to support strict PAs. The reasons why strict PAs are better funded deserves attention, especially if multiple-use PAs are more likely to be located in higher threat areas, as management capacity is likely a strong determinant of conservation success.

Management restrictions are also related to the level of decentralization in our study. We find that strict PAs, and not multiple-use PAs, are more likely to have been decentralized. In other PA evaluation studies, community-managed PAs are more likely to be designated for sustainable uses and so the correlation would run opposite. This underscores that IUCN management restrictions and governance are not equivalent [22], and that impact evaluations that separate out strict versus multiple-use PAs should be more explicit on whether they think they are capturing the influence of PA governance type and or management objectives, on conservation outcomes. We do not find much variation in decentralization level and capacity in this study; thus, we do not find that capacity varies with level of decentralization.

Future research testing the relationships between governance, management and conservation outcomes needs to combine large-N impact evaluations with more attention to theories of change informed through grounded, field-based research. Existing, large-N impact evaluations find considerable heterogeneity in the influence that governance regime, management restrictions, and management capacity have on conservation outcomes. A global assessment suggests that, on average, indigenous and multiple-use areas are just as effective as strict PAs [85], and an Amazon-basin wide study finds no influence of management capacity on PA effectiveness [19]. Large-N studies such as these are needed to tease out interactions across governance and management processes, since they have sufficient heterogeneity to test interaction effects, similar to a recent analysis of PA effectiveness across Mexico that finds that budget has a moderating influence on a PA’s conservation outcomes [16].

These large-N studies need to be coupled, however, with more grounded, field-based research. Currently, the theory linking PA governance and management processes to conservation success is weak. It is possible that a causal relationship exists between some of these factors and this could vary across locations. Qualitative studies are needed to develop better theories on how governance and management are related, and how they moderate conservation success. These field-based studies can also be used to develop measures of governance and management, as done in this study, in order to test their effect using impact evaluation methods. Secondary datasets on management effectiveness do exist for some PAs, but these metrics are not designed for impact evaluation and thus far their correlation with conservation outcomes has been weak [41]. Our experience constructing governance and management indicators from primary data suggests that PA managers can give realistic assessments about decision-making within the PA, the level of administrative support from central authorities, and are eager to provide information to help improve their own management outcomes.

## Conclusion

An increasing number of PA impact evaluations are helping inform whether PAs lead to conservation outcomes. However, most existing PA evaluations do not shed light on why or how PAs lead to these outcomes, with an important gap in linking these changes to governance or management processes [22, 41]. In this paper, we use primary data to develop governance and management indicators and to measure NDVI values (a proxy of ecosystem functioning and deforestation) over a 30-year period in an understudied region of Central America [50]. We find that PAs designated for strict and multiple-use conservation management objectives are achieving positive conservation outcomes in the region. Both centralized and decentralized PAs in Trifinio are effective at maintaining vegetation and enhancing ecosystem function, suggesting that the actors in charge have less impact than other governance and management factors on conservation outcomes. Capacity has the largest influence on whether a PA leads to positive conservation outcomes in our study, with low capacity PAs experiencing losses in NDVI over the 30-year period, which suggests deforestation. The conservation community would benefit from more research that combines large-N samples with field-based research that develops and tests specific theories of change linking governance, management, and conservation outcomes.

## Supporting information

S1 Appendix (DOCX)

S2 Appendix (DOCX)

S1 Table (DOCX)

S2 Table (DOCX)

S3 Table (DOCX)

S1 Fig (DOCX)
