# Supplementary material for: The impact of protected area governance and management capacity on ecosystem function in Central America
Source: PLoS One. 2018 Oct 18;13(10):e0205964. doi: 10.1371/journal.pone.0205964 (PMC6193709; doi:10.1371/journal.pone.0205964)
Supplement: S1 Appendix — (DOCX) [file pone.0205964.s001.docx]

- - - 1. S1 Appendix
      2. Cluster analysis method

We conducted several tests to classify PAs by their level of decentralization and management capacity using several hierarchical cluster methods (Ward’s, Average, Single and Complete linkages) and variable standardization combinations. We dropped all variables with more than two missing values in any observation. We then ran a matrix dissimilarity with Gower procedure using the selected variables and range standardization. In general, most of the solutions from the cluster methods were very similar but not exactly the same which can be expected. Given this result and as suggested by the literature, we decided to proceed only using Ward’s linkage with Gower on the matrix dissimilarity because it preforms better for this type of analysis than the alternative method and it also allows for use of missing values in the observations.

In our cluster analysis, we identified patterns in data based on similarities of observations. Cluster methods are useful in assessing whether or not observations that resemble each other can be summarized in relatively small number of clusters which are different in some way from other clusters [1, 2]. We used detected similarities in the data to create the cluster groups based on relevant questions and hierarchical relationships [3]. The hierarchical clustering techniques is based on compactness where members of each cluster should be as close to each other as possible—the variance is minimized—and separation where the clusters themselves are widely spaced. The most commonly used methods in hierarchical clustering include: Single-linkage (nearest neighbor); Complete-linkage (furthest neighbor); Average-linkage (average distances between all pairs of the two clusters’ members); and Ward’s-linkage (based on the size of sum-of-squares error criterion)—see [1] for a details on each of these method. Also, hierarchical methods are suitable for binary data and for categorical data that can be converted to binary format; some of observation were recorded as binary responses or categorical.

We used the Ward’s method because it tends to find same-size, spherical clusters but is sensitive to outliers [1, 4]. Finally, Ward’s and Average-linkage are appropriate methods to use with continuous data—treating ordered data as continuous by standardizing based on range—as well as using Gower’s similarity measure for mixed data. The use of dissimilarity matrix is advised as an initial measure of concurrency in homogeneity or differentiation in groups and observations. The results of the dissimilarity matrix can then be used in a hierarchical clustering method [1].

Solutions from cluster analysis are intended for generating rather than testing hypotheses and are visually displayed as a tree diagram known as a dendrogram which aids in the selection of clusters that are optimal for the research [3]. We selected the number of clusters generated from observations in the dataset based on stopping rules like Caliński and Harabasz pseudo-F index and the Duda–Hart Je(2)/Je(1) index in which values— larger or smaller depending on the rule—indicate more distinct clustering. Large values of the Caliński –Harabasz pseudo-F index indicate distinct clustering. A large Je(2)/Je(1) index value and a small pseudo-T-squared value indicate distinct clustering [5]. While the former can be used in hierarchical and non-hierarchical clustering the latter work only with a hierarchical cluster analysis [5]. In addition, we used a dendrogram (or tree diagram) which is a graphic mathematical representation of the complete clustering procedure showing the particular partitions or clusters. Each observation conforms a unique cluster at the bottom of the dendrogram. Each observation has a vertical line connecting other observations with a horizontal line. Horizontal lines define clusters and the vertical distances show (dis)similarity values and the distance of the lines indicate more or less distinct separation between the groups—shorter vertical lines indicate similarity. The groupings continue and end at the top of the dendrogram where all observations conform a single cluster. The number of clusters to select from a dendrogram is based on the differences in height (y-axis) where a horizontal partition across all observations determine that clusters below that height are (dis)similar from each other at the value of the difference in height from other horizontal partitions [5]. Thus, the graphic display of the dendrogram can informally suggest the number of clusters to select—the large change in height indicates the best horizontal cut. [1].

- - - 1. References

1. Everitt BS, Landau S, Leese M, Stahl D. Cluster analysis. 5 ed. West Sussex, United Kingdom: John Wiley & Sons, Ltd.; 2011.

2. Rencher AC, Christensen WF. Methods of multivariate analysis. Hoboken, N.J.: Wiley; 2013.

3. Hamilton LC. Statistics with STATA: Version 12. Boston, MA: Cengage Learning; 2012.

4. Rabe-Hesketh S, Everitt B. A Handbook of statistical analyses using Stata. 3 ed: Chapman & Hall/CRC Press; 2004.

5. StataCorp. Stata base reference manual 14. College Station, TX: StataCorp; 2015.
