# Supplementary material for: The impact of protected area governance and management capacity on ecosystem function in Central America
Source: PLoS One. 2018 Oct 18;13(10):e0205964. doi: 10.1371/journal.pone.0205964 (PMC6193709; doi:10.1371/journal.pone.0205964)
Supplement: S2 Appendix — (DOCX) [file pone.0205964.s002.docx]

- - - 1. S2 Appendix
      2. NDVI classification

Medium resolution remotely sensed Landsat images for Trifinio were acquired from the USGS Earth Resources and Observation Science Center (USGS/EROS). Surface reflectance values were used develop least-cloud vegetation-index based on greenness to measure vegetation disturbance and regrowth, as a proxy to measure the extent of vegetation greenness change, over approximately five-year epochs. We used greenness as the metric for transition rather than land cover class type, due to the spectral similarity between coffee and agroforestry systems [1, 2]. We used a single Landsat scene (World Reference System (WRS) Path/Row 19/50) per epoch that comprises the Trifinio Region. The source data had already been orthocorrected, and radiometrically and atmospherically corrected [3] and processed to a cloud-shadow Fmask [4, 5]. Fmask data were re-classed to Boolean layers—for USGS/EROS.

As these data are passively-sensed and cloud cover is an issue in the region, least cloud data are only feasibly accessible during the dry season (between January and March). The cloud and shadow Fmask Boolean layers were used to mask out the compromised image sectors from all processing and analyses streams. After cloud masking, all raster images and vector data files were resampled to a common raster grid using the Universal Transverse Mercator (UTM) Zone 16N using the WGS1984 datum, while maintaining the original Landsat resolution of 30 meters, using remotely sensed image processing tools (ArcGIS, v. 10.3; ENVI “ENvironment for Visualizing Images” v. 5.3.1; and TerrSet v.18.21; TerrSet’s SAMPLE.exe). NDVI layers were prepared to create ranked greenness in quintile-based categories [6]. When more than one image made up an epoch, least cloud images were formed by combining them into epochs using maximum NDVI value criteria. For example, the maximum value of NDVI will be the output of three very cloudy images, only after all clouds and shadows have been removed by masking. Epochs may be made up of data from an adjacent year if it is in the same dry season period, nearest the month of March. Pixels classified as water were combined in a single water mask to remove all water pixels across all images to prevent water-related false transitions. The original range of Surface Reflectance (SR) data were from -10,000 to +20,000 (scaled to Real reflectance values with a factor of 0.0001), however valid data are only held in those values between -10,000 and +10,000, and thus data were capped at 0 and +10,000 to assess greenness.

- - - 1. References

1. Cordero‐Sancho S, Sader SA. Spectral analysis and classification accuracy of coffee crops using Landsat and a topographic‐environmental model. International Journal of Remote Sensing. 2007;28(7):1577-93.

2. Schmitt-Harsh M. Landscape change in Guatemala: Driving forces of forest and coffee agroforest expansion and contraction from 1990 to 2010. Applied Geography. 2013;40:40-50. doi: <http://dx.doi.org/10.1016/j.apgeog.2013.01.007>.

3. Masek JG, Vermote EF, Saleous N, Wolfe R, Hall FG, Huemmrich KF, et al. LEDAPS Calibration, Reflectance, Atmospheric Correction Preprocessing Code, Version 2. 2013. doi: 10.3334/ORNLDAAC/1146.

4. Zhu Z, Woodcock CE. Object-based cloud and cloud shadow detection in Landsat imagery. Remote Sensing of Environment. 2012;118:83-94. doi: 10.1016/j.rse.2011.10.028.

5. USGS. User guide: earth resources observation and science (EROS) center science processing architecture (ESPA) on demand interface. USA: U.S. Geological Survey; 2016.

6. Rouse JW, Haas RH, Schell JA, Deering DW. Monitoring the Vernal Advancement and Retrogradation (Green Wave Effect) of Natural Vegetation. Progress Report. Greenbelt, MD: NASA GSFC, 1973 1973. Report No.
