# Supplementary material for: The impact of protected area governance and management capacity on ecosystem function in Central America
Source: PLoS One. 2018 Oct 18;13(10):e0205964. doi: 10.1371/journal.pone.0205964 (PMC6193709; doi:10.1371/journal.pone.0205964)
Supplement: S1 Table — (DOCX) [file pone.0205964.s003.docx]

- - - 1. S1 Table
      2. Table 1. Summary of interview questions themes used for PA governance and management capacity classification

| **Decentralization interview questions** | |
| --- | --- |
| **Theme** | **Coded response** |
| Entity responsible for PA (e.g., Secretary or Ministry) | 1 = If one central government agency is responsible for PA  0 = If organization co-manager with government agency are responsible for PA. |
| Appointment of director or person responsible for PA | 1 = A central authority appoints the person in charge of the PA  0 = If not |
|  | 1 = Central authority appoints the person in charge of the PA with local consultation  0 = If not |
|  | 1 = A local authority appoints the person in charge of the PA in consultation with a central authority  0 = if not |
| Ways this person makes management decisions for PA | 1 = Ordinary daily decisions are made locally and certain general decisions need consultation with central office  0 = If decisions are only made at central office |
| Participation | The number of stakeholders involved in decision-making and management activities, and whether this number has increased or decreased over time. This includes number of actors, actors’ perceptions about the PA, and the involvement of actors in decision-making and management. |
| Frequency of meetings with external relevant actors | 1 = Usually monthly meetings or more according to the need/issue in hand  0 = Never. The magnitude of the relation measured by meetings and the number of years since relevant actors started to meet. |
| Existence of co-management agreements | 1 = If there is co-management agreement  0 = If not |
| External actors (stated quantity) | Quantity stated or counted |
| Co-manager | Number of co-managers. The level of decentralization is measured by the existence of co-management agreements and the number of actors involved in management. |
| Likert scale responses on decentralization | Sum of responses of nine Likert statements related to decentralization with higher values indicating more decentralization. |
| **Management capacity interview questions** | |
| **Theme** | **Coded response** |
| Existence of written management plan | 1 = If there is written management plan  0 = otherwise |
| Existence of written annual operations plan | 1 = If there is an annual operations plan  0 = otherwise |
| Main sources of funding for PA | 1 = National Budget is the main source of funding  0 = Occasionally there is funding from Trifinio Commission projects |
| Year to year budget fluctuation | 1 = Budget does not fluctuate from year to year  0 = Budget fluctuates from year to year mainly with funds from projects |
| Is current staff enough | 1 = If staff is not sufficient for the administration and management of the PA  0 = Otherwise |
| Data about PA is generated and available | 1 = If some data about PA is available  0 = If not |
| Distribution of budget in percentages | Percentage of budget for salaries |
| Total paid staff | Number of paid staff |
| Likert scale responses on capacity | Sum of responses of seven Likert statements related to management capacity with higher values indicating more capacity. |
