# Supplementary material for: The impact of protected area governance and management capacity on ecosystem function in Central America
Source: PLoS One. 2018 Oct 18;13(10):e0205964. doi: 10.1371/journal.pone.0205964 (PMC6193709; doi:10.1371/journal.pone.0205964)
Supplement: S2 Table — (DOCX) [file pone.0205964.s004.docx]

- - - 1. S2 Table
      2. Table 1. Post-matching OLS regression output for average annual change in NDVI

| **Variable** | **Strict PA** | **Multiple-use PA** | **High Capacity** | **Low Capacity** | **High Decentralization** | **Low Decentralization** |
| --- | --- | --- | --- | --- | --- | --- |
| Protected area | 0.053*** | 0.055*** | 0.090*** | -0.068*** | 0.082*** | 0.051*** |
|  | (0.005) | (0.005) | (0.005) | (0.008) | (0.007) | (0.004) |
| 1986 NDVI value | 1.133*** | -1.448*** | -1.375*** | -1.275*** | -1.062*** | -1.326*** |
|  | (0.024) | (0.025) | (0.019) | (0.034) | (0.033) | (0.018) |
| Elevation (masl) | 0.00006*** | 0.0002*** | 0.00006*** | 0.0005*** | -0.0001*** | 0.0002*** |
|  | (0.00001) | (9.92e-06) | (8.41e-06) | (0.0002) | (0.00002) | (8.69e-06) |
| Slope (%) | 0.002*** | 0.002*** | 0.001*** | 0.001*** | 0.001*** | 0.001*** |
|  | (0.0001) | (0.0001) | (0.0001) | (0.0002) | (0.0001) | (0.0001) |
| Distance to road (km) | 0.01*** | 0.01*** | 0.009*** | 0.003 | 0.01*** | 0.01*** |
|  | (0.001) | (0.001) | (0.001) | (0.003) | (0.001) | (0.001) |
| Distance to municipal capital (km) | 0.001** | 0.001* | 0.0004 | -0.0009 | 0.0003 | 0.002*** |
|  | (0.001) | (0.001) | (0.0004) | (0.001) | (0.001) | (0.0004) |
| Distance to country capital (km) | 0.0009*** | 0.002*** | 0.001*** | -0.0006*** | 0.0007*** | 0.002*** |
|  | (0.0001) | (0.0001) | (0.0001) | (0.0002) | (0.0001) | (0.00004) |
| R2 | 0.18 | 0.27 | 0.25 | 0.23 | 0.15 | 0.25 |
| Observations | 14,500 | 20,292 | 22,714 | 6,902 | 7,784 | 25,384 |

Note: Mean values with standard errors in parentheses.

- - - 1. Table 2. Post-matching OLS regression output for mean NDVI value

| **Variable** | **Strict PA** | **Multiple-use PA** | **High Capacity** | **Low Capacity** | **High Decentralization** | **Low Decentralization** |
| --- | --- | --- | --- | --- | --- | --- |
| Protected area | 0.020*** | 0.017*** | 0.027*** | -0.015*** | 0.028*** | 0.016*** |
|  | (0.001) | (0.001) | (0.001) | (0.002) | (0.001) | (0.001) |
| 1986 NDVI value | 0.706*** | 0.681*** | 0.667*** | 0.739*** | 0.706*** | 0.703*** |
|  | (0.004) | (0.004) | (0.003) | (0.006) | (0.005) | (0.003) |
| Elevation (masl) | 9.06e-06*** | 0.00004*** | 1.67e-06 | 0.00009*** | -0.00003*** | 0.00003*** |
|  | (1.89e-06) | (1.73e-06) | (1.48e-06) | (3.14-06) | (4.11e-06) | (1.56e-06) |
| Slope (%) | 0.0003*** | 0.0003*** | 0.0003*** | 0.0002*** | 0.0003*** | 0.0002*** |
|  | (0.00002) | (0.00002) | (0.00002) | (0.00004) | (0.00003) | (0.00002) |
| Distance to road (km) | 0.003*** | 0.005*** | 0.002*** | 0.003*** | 0.003*** | 0.005*** |
|  | (0.0002) | (0.0003) | (0.0002) | (0.0006) | (0.0003) | (0.0003) |
| Distance to municipal capital (km) | -0.0001 | 0.00001 | -0.0003*** | -0.0009*** | -0.0001 | 0.0002** |
|  | (0.0001) | (0.0001) | (0.0001) | (0.0002) | (0.00001) | (0.0001) |
| Distance to country capital (km) | 0.0002*** | 0.0001*** | 0.0003*** | 0.0002*** | 0.0001*** | 0.0005*** |
|  | (9.86e-06) | (9.26e-06) | (8.69e-06) | (0.00003) | (0.00005) | (8.16e-06) |
| R2 | 0.71 | 0.78 | 0.72 | 0.82 | 0.73 | 0.76 |
| Observations | 14,500 | 20,292 | 22,714 | 6,902 | 7,784 | 25,384 |

Note: Mean values with standard errors in parentheses.
