# Supplementary material for: The impact of protected area governance and management capacity on ecosystem function in Central America
Source: PLoS One. 2018 Oct 18;13(10):e0205964. doi: 10.1371/journal.pone.0205964 (PMC6193709; doi:10.1371/journal.pone.0205964)
Supplement: S3 Table — (DOCX) [file pone.0205964.s005.docx]

- - - 1. S3 Table
      2. Table 4. Covariate balance checks following matching PA observations to not PA observations

| **Variable** | **Strict PA** | | **Multiple-use PA** | | **High Capacity** | | **Low Capacity** | | **High Decentralization** | | **Low Decentralization** | |
| --- | --- | --- | --- | --- | --- | --- | --- | --- | --- | --- | --- | --- |
|  | %QQ | Mean Diff | %QQ | Mean Diff | %QQ | Mean Diff | %QQ | Mean Diff | %QQ | Mean Diff | %QQ | Mean Diff |
| Elevation (masl) | 96.84 | 0.09 | 98.12 | 0.04 | 96.5 | 0.10 | 99.94 | 8.0e-04 | 98.77 | 0.09 | 99.93 | 0.001 |
| Slope (%) | 96.95 | 0.02 | 92.87 | 0.04 | 99.12 | 0.01 | 99.97 | 1.9e-04 | 96.06 | 0.02 | 97.1 | 0.02 |
| Distance to road (km) | 85.09 | 0.09 | 908.1 | 0.02 | 85.41 | 0.06 | 119.5 | 0.08 | 69.37 | 0.21 | 20.42 | 0.02 |
| Distance to municipal capital (km) | 79.45 | 0.03 | 33.56 | 0.04 | 89.88 | 0.01 | 75.91 | 0.10 | 89.49 | 0.01 | -52.5 | 0.01 |
| Distance to country capital (km) | 69.84 | 0.06 | 26.83 | 0.09 | 88.05 | 0.05 | 99.16 | 0.02 | 98.61 | 0.01 | 67.21 | 0.07 |

*Note: “%QQ” refers to the percent reduction in mean deviation of quantile-quantile plots between treatment and control observations after matching the protected areas to control observations. “Mean Diff” refers to the normalized difference in mean, which is the difference in means as a proportion of the standard deviation. A value less than 0.25 is considered sufficient for removing bias between treatment and control groups* [1].

- - - 1. References

1. Imbens GW, Wooldridge JM. Recent Developments in the Econometrics of Program Evaluation. Journal of Economic Literature. 2009;47(1):5-86. doi: doi: 10.1257/jel.47.1.5.
