# Supplementary material for: The impact of protected area governance and management capacity on ecosystem function in Central America
Source: PLoS One. 2018 Oct 18;13(10):e0205964. doi: 10.1371/journal.pone.0205964 (PMC6193709; doi:10.1371/journal.pone.0205964)
Supplement: S1 Fig — (DOCX) [file pone.0205964.s006.docx]

- - - 1. S1 Figure

- - - 1. Figure 1. Trends in NDVI values by PA sub-groups compared to non-PA pixels.

1. Level of Restriction

1. Level of Management Capacity
2. Level of Decentralization

Figure 1. Trends in NDVI values by PA sub-groups compared to non-PA pixels.
